# Longitudinal survey of malaria morbidity over 10 years in Saharevo (Madagascar): further lessons for strengthening malaria control

**DOI:** 10.1186/1475-2875-8-190

**Published:** 2009-08-06

**Authors:** Léon P Rabarijaona, Milijaona Randrianarivelojosia, Lucie A Raharimalala, Arsène Ratsimbasoa, Arthur Randriamanantena, Laurence Randrianasolo, Lanto A Ranarivelo, Fanja Rakotomanana, Rindra Randremanana, Jocelyn Ratovonjato, Marie-Ange Rason, Jean-Bernard Duchemin, Adama Tall, Vincent Robert, Ronan Jambou, Frédéric Ariey, Olivier Domarle

**Affiliations:** 1 Institut Pasteur de Madagascar, BP 1274 Antananarivo (101), Madagascar; 2 UNICEF, Antananarivo, Madagascar; 3 RTI/SanteNet2, Fort Duschesne, Antananarivo (101), Madagascar; 4 Service de Lutte contre le Paludisme, Ministère de la Santé et du Planning Familial, Antananarivo (101), Madagascar; 5 CERMES, Niamey, Niger; 6 Institut Pasteur de Dakar, Sénégal; 7 Institut de Recherche pour le Développement UR-016 et Muséum National d'Histoire Naturelle USM-504, Centre IRD de Montpellier, B.P. 64501, 34394 Montpellier cedex 5, France; 8 Institut Pasteur, Département de Parasitologie Mycologie Paris, France; 9 Institut Pasteur du Cambodge, Phnom Penh, Cambodge

## Abstract

**Background:**

Madagascar has been known for having bio-geo-ecological diversity which is reflected by a complex malaria epidemiology ranging from hyperendemic to malaria-free areas. Malaria-related attacks and infection are frequently recorded both in children and adults living in areas of low malaria transmission. To integrate this variability in the national malaria control policy, extensive epidemiological studies are required to up-date previous records and adjust strategies.

**Methods:**

A longitudinal malaria survey was conducted from July 1996 to June 2005 among an average cohort of 214 villagers in Saharevo, located at 900 m above the sea. Saharevo is a typical eastern foothill site at the junction between a costal wet tropical area (equatorial malaria pattern) and a drier high-altitude area (low malaria transmission).

**Results:**

Passive and active malaria detection revealed that malaria transmission in Saharevo follows an abrupt seasonal variation. Interestingly, malaria was confirmed in 45% (1,271/2,794) of malaria-presumed fevers seen at the health centre. All four *Plasmodia *that infect humans were also found: *Plasmodium falciparum*; *Plasmodium vivax*, *Plasmodium malariae *and *Plasmodium ovale*. Half of the malaria-presumed fevers could be confirmed over the season with the highest malaria transmission level, although less than a quarter in lower transmission time, highlighting the importance of diagnosis prior to treatment intake. *P. falciparum *malaria has been predominant (98%). The high prevalence of *P. falciparum *malaria affects more particularly under 10 years old children in both symptomatic and asymptomatic contexts. Children between two and four years of age experienced an average of 2.6 malaria attacks with *P. falciparum *per annum. Moreover, estimated incidence of *P. falciparum *malaria tends to show that half of the attacks (15 attacks) risk to occur during the first 10 years of life for a 60-year-old adult who would have experienced 32 malaria attacks.

**Conclusion:**

The incidence of malaria decreased slightly with age but remained important among children and adults in Saharevo. These results support that a premunition against malaria is slowly acquired until adolescence. However, this claims for a weak premunition among villagers in Saharevo and by extension in the whole eastern foothill area of Madagascar. While the Malagasy government turns towards malaria elimination plans nowadays, choices and expectations to up-date and adapt malaria control strategies in the foothill areas are discussed in this paper.

## Background

In Madagascar island, a broad range of malaria transmission patterns coexist following the local bio-geo-ecological diversity. In coastal areas, high levels of malaria transmission prompt the development of acquired immunity [1-3]. Even though life-threatening malaria such as cerebral malaria is relatively rare in these regions, the incidence of severe anaemia is high and especially affects children. In contrast, seasonal and low malaria transmission pattern are typical of highland plateaus located at 1,000 to 1,500 meters above the sea. Acquired immunity is low there and malaria affects all age groups [4,5]. Between highlands and coasts, the complexity of malaria transmission patterns increases. Depicted as an autonomous transmission area, intermediate zones might be responsible of parasites re-invasion in the highlands. Unfortunately, epidemiologic characteristics remain poorly understood in these areas although understanding and controlling the malaria parasite transmission there appear crucial to eliminate malaria in Madagascar. To get a more accurate definition of malaria transmission in such intermediate areas, an epidemiological study was performed among the villagers of Saharevo. Data collected from July 1996 to June 2005 were the basis of an extensive study giving rise to an in-depth local surveillance reported herein which also highlights key points to define and adapt malaria control strategies.

## Methods

### Biogeography and malariometric parameters at study site

**Figure 1:**
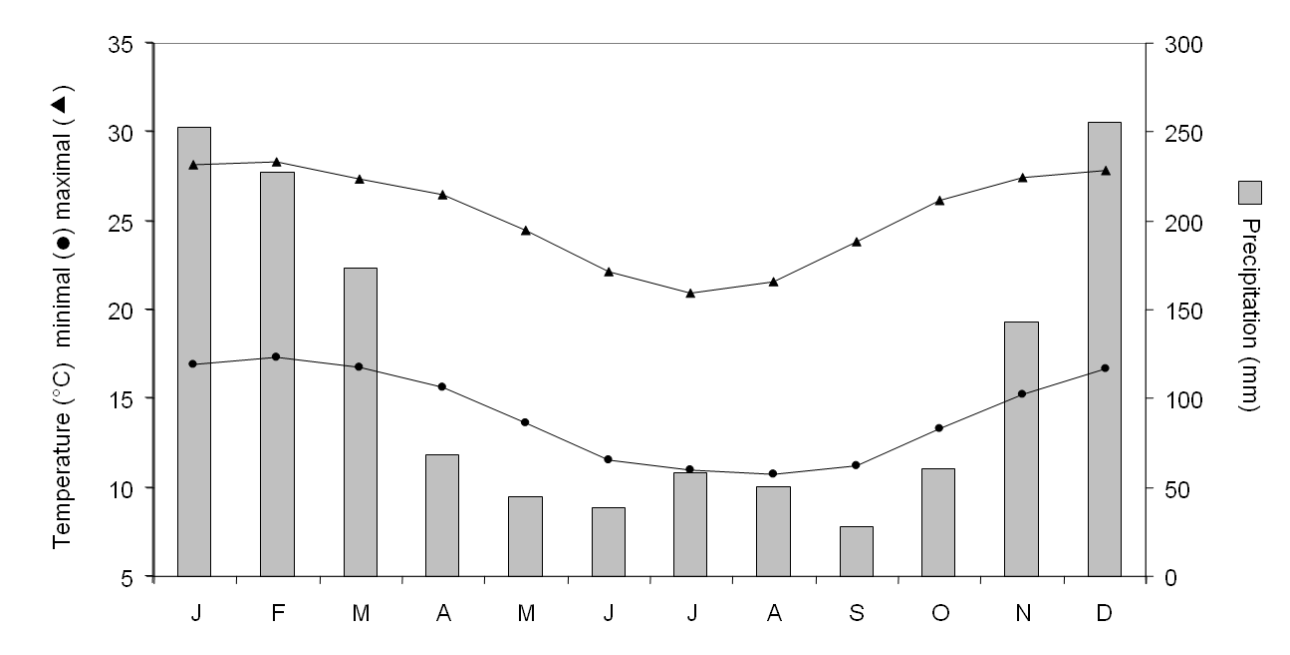
**Climatic data from Moramanga, 12 km away from Saharevo**. Profiles extracted from data collected over the past 30 years (1961–1990).

Preliminary surveys in 1995 pointed Saharevo as a typical intermediate epidemiological site according to the criterions of malaria endemicity defined by the Ministry of Health and Family Planning [1]. Located in the eastern foothills of Madagascar, 100 km East of Antananarivo (latitude 18°82'S, longitude 48°10'E, altitude 900 meters) the village is properly set up at the boundary between the east coastal and the highlands areas. Also, thanks to its intermediate geographical location, Saharevo is characterized by climate and biotope just at the interface between the more extreme faces observed in the highlands and the eastern coast. Temperature and rainfall patterns were recorded at the nearest meteorological station at Moramanga (12 km from Saharevo). Profiles extracted from data collected over the past 30 years revealed the existence of a five month rainy season with heat and precipitation peaks from November to March (Figure 1). Besides a geographical definition adapted to the study of intermediate areas, no indoor residual spraying of insecticides, no chemoprophylaxis, no intermittent preventive treatment was ever conducted in Saharevo before 2006 but only limited use of bed nets. Entomological investigation showed the simultaneous presence of four malaria-parasites vectors: *Anopheles funestus*, *Anopheles mascarensis*, *Anopheles gambiae *and *Anopheles arabiensis*. Malaria transmission assessment performed between October 2003 and September 2004 revealed an annual entomological inoculation rate (EIR) of 2.78 per adult. *An. funestus *mediated approximately 75% of the transmission that occurred from February to May. (Andrianaivolambo and colleagues, in preparation)

### Study cohort

Saharevo annual population census between 1996 and 2005 rose from 182 to 261 inhabitants, with a gap of data in 2002 due to post-electoral turmoil. A mean of 214 inhabitants per annum was estimated and on average half of them were less than 15 years old. This is typical of developing countries and of Madagascar. Due to regular neighborhood communities visits at Saharevo, a total of 325 participants were included in this study. Two main ethnic groups, Bezanozano and Merina, coexist in the village and form 43 families distributed among 39 houses built according to the traditional style of mud walls and thatched roofs. Major activities rely on charcoal production and agricultural works: essentially rice culture during the rainy season from November to April, coupled with local production of cassava, peanuts, sweet potatoes, and garden vegetables. Small herds of domestic animals grew near the villagers. About 50 beef cattle lived in enclosures at the vicinity of houses while fowl roamed free in the daytime though kept home, usually in the kitchen, over night. Short time adult migrations to areas with distinct malaria transmission compared to Saharevo profile regularly occurred but without any defined frequency.

### Clinical and parasitological monitoring

Villagers were enrolled to the survey on a consent basis. After the first round census, a unique code number was attributed to any participant for basic demographic and socio-economic indicators to be later confronted with the clinical and biological data that would be continuously collected during the project. The follow-up consisted in malaria case detection on a monthly active basis among villagers and in a passive way among outpatients visiting the dispensary, where a physician was present 24 h a day, five days a week. The clinical signs and symptoms associated with malaria attacks were systematically recorded following an ad hoc questionnaire and recorded in patient personal folders. Each examination was completed with measurement of axillary temperatures with digital thermometers and collection of blood smears from finger pricks.

At each visit, fingerpick blood samples were collected for parasitological checking. Thin and thick smears were carried out on glass slides and stained with Giemsa. Two samples of blood smears were systematically made for each patient. In case of malaria suspicion, one slide was immediately examined to confirm or not the diagnosis. All blood smears were examined by experienced physicians or technicians. *Plasmodium falciparum *densities were calculated after counting asexual and sexual parasites among 1,000 leukocytes, further converted into parasites load (trophozoits and/or gametocytes) per microliter of blood. Assuming an average leukocyte count of 8,000/μl (WHO, 1991), the detection threshold was about eight parasites/μl of blood. The gametocytes density was separately recorded. Blood smears were stored at the Intitut Pasteur de Madagascar in Antananarivo for quality control purposes.

### Passive malaria detection

Microscopic passive malaria detection was done at the primary health centre at Saharevo in any patients with fever (axillary temperature ≥37.5°C) or reporting a recent fever history. Presence of asexual *Plasmodium *confirmed malaria attack. In that case, patient were given a chloroquine treatment – as front line treatment – in agreement with the national malaria control policy guidelines [6-8]. Chloroquine was administered under physician supervision at 25 mg/kg over three days (10 mg/kg during the two first days and 5 mg/kg the third day). In 2005, chloroquine was left and amodiaquine was used at 10 mg/kg/day over three days. Paracetamol was simultaneously administered for three days at 50 mg/kg/day. Malaria attacks recurring within 31 days were considered as recrudescent infections and treated with the second line treatment (sulphadoxine-pyrimethamine). When microscopy did not confirm malaria or in case of non-malaria pathologies, symptomatic or specific treatment was administered according to the national health policy. Finally, the population was asked to report to the physician any other drug intakes.

### Active malaria detection

Every study participant underwent a monthly examination including measurement of axillary temperature and blood smears collection for active malaria detection by microscopy. Interest of systematic screening relies on the surveillance of both symptomatic and asymptomatic parasite carriers. Patients with presence of asexual parasites in blood but without fever (axillary temperature <37.5°C) were reported asymptomatic carriers. Data were collected from February 2003 to June 2005.

### Data management and analysis

Data were double-entered into Microsoft Access^® ^version 2.6 databases (Microsoft Corporation) and completeness and internal integrity checks performed on a weekly basis. Analysis was done using SPSS^® ^version 10.0 (Stata Corp., College Station, TX, USA) according to a pre-agreed analytical plan. Statistical significance was set at *P *< 0.05.

### Ethical considerations

Authorizations to carry out malaria studies in Saharevo were delivered by the Ministry of Health and Family Planning (MoH), the Regional Direction of Health in Mangoro, the Health District in Moramanga, and by local administrative officials. Ethical clearance was obtained from the national ethical committee. Also, to ensure and optimize villagers' commitment to the project, information meetings were regularly held in Saharevo providing explanation related to the protocol and the objectives of this study in Malagasy language. Individual informed consent was obtained from each participant or legal guardians for children. Participation in the project was on a voluntary basis, and all villagers were eligible without exclusion criterion. Primary health care was provided free of charge to everyone, including those who did not join the cohort. The Institut Pasteur de Madagascar is among the major technical partners of the MoH. Saharevo is part of the national network for the surveillance of malaria parasite resistance to drugs – a network known as RER for Réseau d'Etude de la Résistance, recognized by the MoH since 1999 [9-11].

## Results

### High seasonality of malaria infection in Saharevo

**Figure 2:**
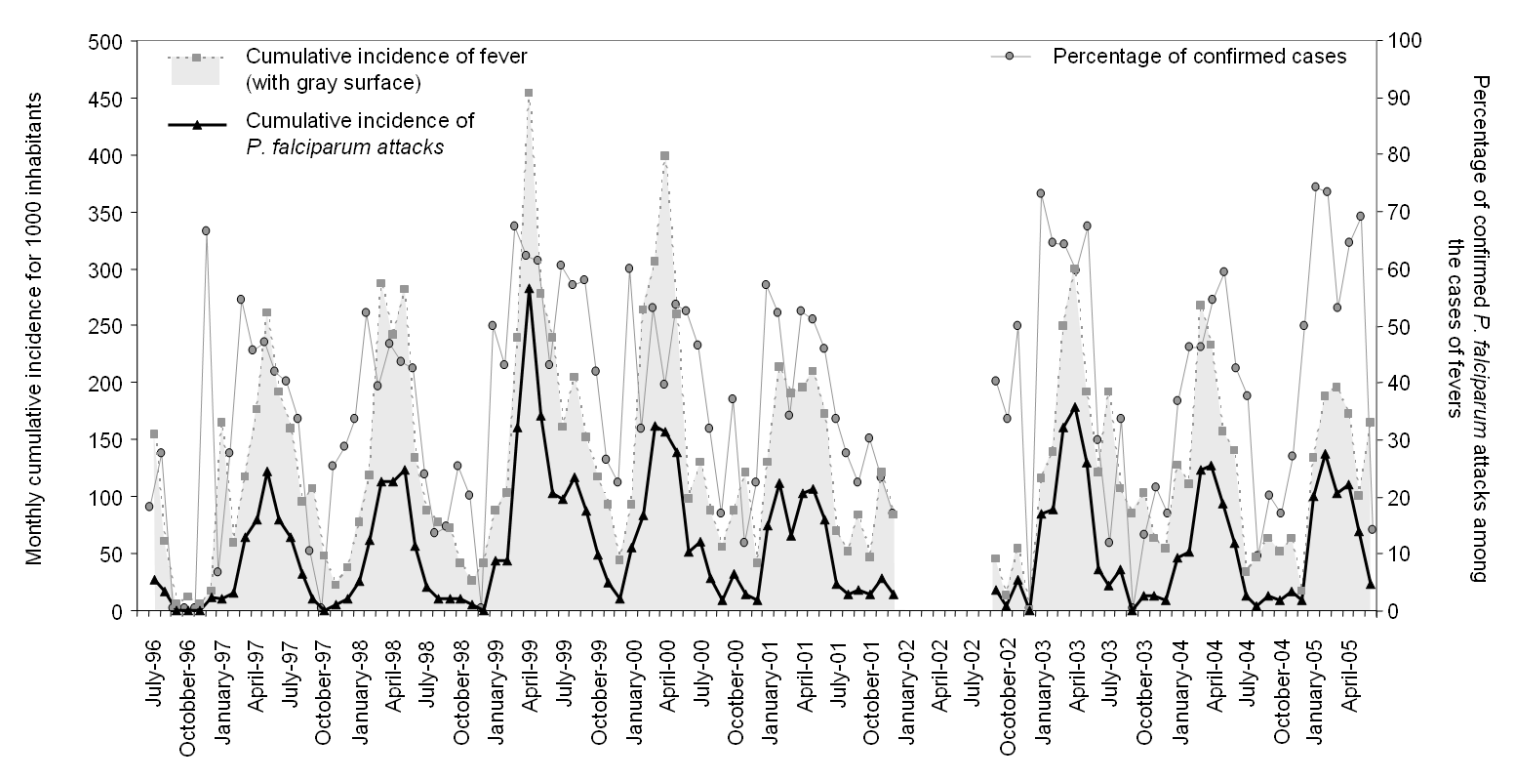
**Monthly incidence of fever (uncorrected axillary temperature ≥37.5°C) and *P. falciparum *malaria attacks (fever + asexual parasites) for 1,000 inhabitants**. According to the annual censuses: 182 inhabitants in 1996, 188 (1997), 195 (1998), 205 (1999), 216 (2000), 215 (2001), not done in 2002, 224 (2003), 236 (2004), and 261 (2005). The average monthly incidence of malaria *P. falciparum *attacks was 5.6%.

A total of 9,556 medical consultations were recorded at the primary health centre at Saharevo from July 1996 to June 2005. Passive malaria case detection consisted in examination and diagnosis of every outpatient presenting or reporting fever. On this basis, among 2,794 (29.2%) cases of presumed malaria, 1,271 (45.5%) malaria attacks were confirmed by microscopy. All four *Plasmodia *that infect humans were detected: 1,241 (97.6%) *P. falciparum*, 24 (1.9%) *P. vivax*, including two (0.2%) mixed infections with *P. falciparum*, eight cases of *P. malariae *(0.6%), including two (0.12%) mixed infections with *P. falciparum*, and two cases of *P. ovale *(0.2%). While *P. falciparum *appeared as the likeliest species responsible of malaria attacks, it is also worth notice that 15% (n = 186) of those episodes occurred in less than 31 days after a previous attack in the same patient. This indicates that chloroquine treatment failed and such failure demanded a second line treatment. Annual analysis over 10 year time of the study highlighted how strongly seasonal the incidences of fever and malaria attack are. Indeed, similar patterns of incidence have been recorded for both parameters with an annual recurrence. Remarkably a peak incidence of symptomatic *P. falciparum *malaria occurred in April/May even though amplitude variation was noticed from one year to another (Figure 2). Monthly, the average incidence of *P. falciparum *attacks was 5.6%, and the proportion of confirmed *P. falciparum *malaria among suspected cases was 38.7% (47.2% from January to July and 22.4% in August to December).

Seasonal fluctuations of *P. falciparum *malaria rates were recorded in both children and adults. However several observations tend toward that seasonality might be even more marked in children: (i) in children <15 years, *P. falciparum *malaria attack rate dropped from 55.1% over January – July to 31.6% in August – December (χ^2 ^test *p *< 0.01); although (ii) in adults ≥15 years old, it only dropped from 35.2% to 19.7% over the same time frames (χ^2 ^test *p *< 0.01). Interestingly, men had significantly much more *P. falciparum *malaria attacks than women with 47.7% confirmed malaria cases *vs *41.1% respectively (χ^2 ^test *p *< 0.001). Nevertheless, no sex related difference has been reported for annual incidence (mean annual incidences for men and women: 38.6 ± 13.3% and 33.1 ± 12% respectively).

### *P. falciparum *malaria attacks in Saharevo children

**Figure 3:**
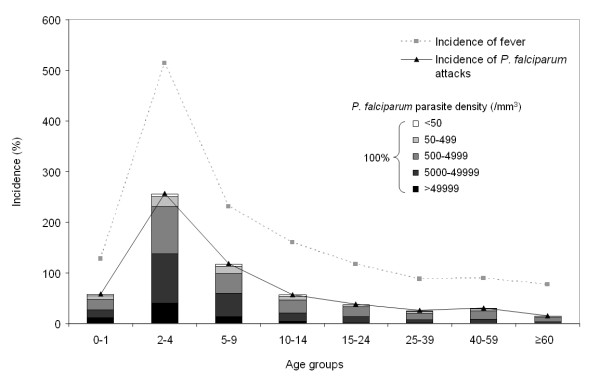
**Curves of the annual age-specific incidence of fever (uncorrected axillary temperature ≥37.5°C) and *P. falciparum *malaria attacks (fever + asexual parasites)**. Histograms represent the parasite densities for *P. falciparum *(proportion of the parasite densities in number of parasites per μl of blood, for 100% of the malaria attacks). Values are drawn from passive monitoring of entire years (1997 to 2004, except 2002). Using only subjects with a recorded age from those years, the average number of inhabitants was 201 (ranged from 153 in 1997 to 236 in 2004). For each sample, the age of the subject used is the age at the time of the blood collection.

Based on census data, annual incidence of fever and *P. falciparum *malaria was estimated according to age groups (Figure 3). A peak incidence of both fever and confirmed malaria attacks is remarkably observed in children from two to four years old. Moreover, among children less than 10 years old, 49.3% of those fevers were confirmed malaria attacks, although only 30.6% among outpatients beyond that age. In accordance with above data, most malaria cases were related to *P. falciparum *and 70.8% of them were recorded in children under 10 years old (843 out of 1,190). The annual cumulative *P. falciparum *malaria incidence also support those observations and displayed 4.33 times rise in children under 10 years old (145% versus 33.5% if over 10 years old). On the top of that, this estimation indicated that children from two to four years old were likely to undergo on average 2.6 malaria attacks per annum, and in general a total of 14.8 malaria attacks before reaching 10 years old. Since the risk goes up to 31.7 malaria attacks by 60 years old, this means that half of malaria attacks were likely to occur during the first 10 years of life. This also indicate that premunition against malaria was gradually acquired during adolescence. In agreement, parasite densities during malaria attacks remained significantly higher in children than adults (Figure 3). In children under 10 years old, parasite densities over 5,000 parasites/μl have been recorded in 50.3% *P. falciparum *related attacks although only 28.5% of adolescents and adults (χ^2 ^test p < 0.01). Altogether this reinforces that young children may be more vulnerable to *Plasmodium *in Saharevo area. In accordance with the high seasonality reported above, most attacks (83.8%) with significant parasite density (beyond 50,000 parasites/μl) occurred over January – July.

Finally, as long as the medical team was on site – a team from the Institut Pasteur de Madagascar – no death attributed to malaria was recorded. Unfortunately, three children deaths have been reported in 2002, during the post-electoral turmoil, while medical assistance and project conduction had to be temporary suspended. Verbal autopsy revealed that those children suffered from strong fevers and convulsions.

### *P. falciparum *in asymptomatic infections

**Figure 4:**
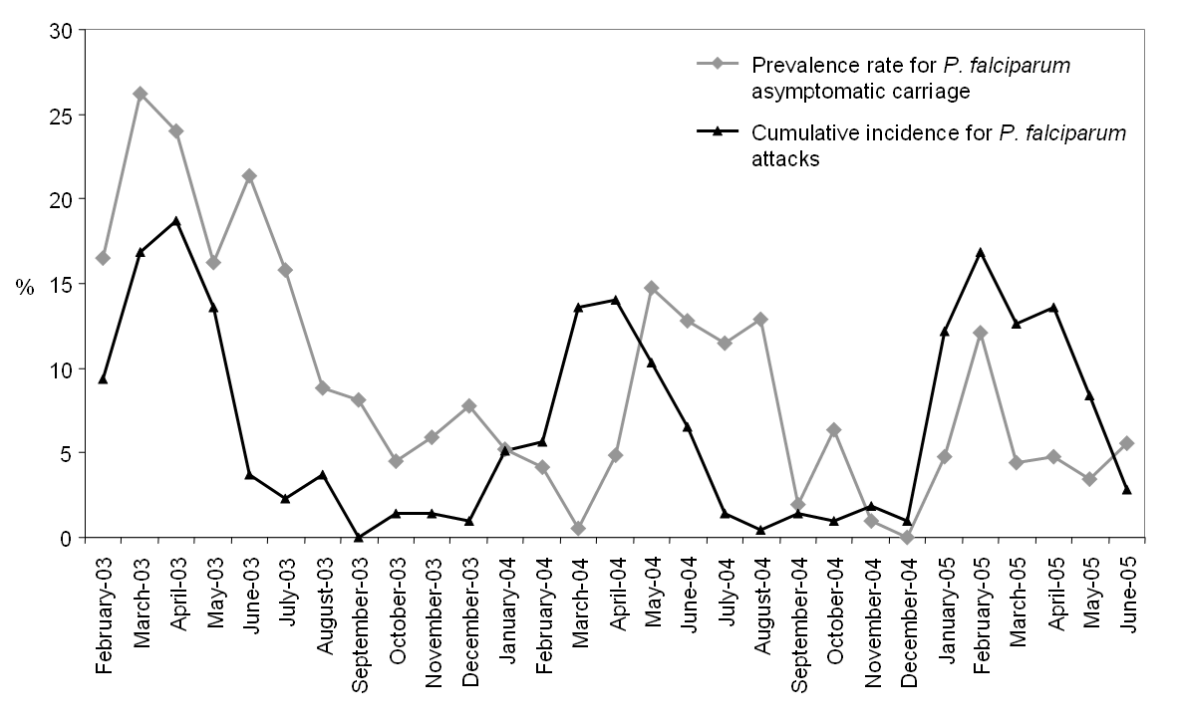
**Monthly prevalence rate (%) for *P. falciparum *measured in active detection cases compared to cumulative *P. falciparum *incidence (Figure 2) from February 2003 to June 2005**.

In contrast with passive malaria detection described above, an active malaria detection survey has been simultaneously conducted in Saharevo, in which the cohort of villagers was systematically examined on a monthly basis. An average of 210 patients was checked each month by the medical team from February 2003 to June 2005. A total of 6,078 blood smears were examined, which correspond to a proportion of 82.8% of the inhabitants with respect to the census figures. Participants were then examined from six and 29 times over a 29-month follow-up. Microscopy could detect the presence of *Plasmodium *sp in 558 (9.2%) blood smears. In accordance with the results from the passive malaria detection, all four *Plasmodia *that infect humans were also found: 541 (97%) *P. falciparum*; 15 (2.7%) *P. vivax*, including one mixed infection with *P. falciparum*; two *P. malariae *(0.4%), and one *P. ovale *(0.2%). Malaria prevalence rate confirmed the annual variations with a peak of the incidence of *P. falciparum *malaria attack on April (Figure 4, black spot). Prevalence of *P. falciparum *asymptomatic carriage (Figure 4, grey plot) was then confronted to the attacks incidence. The rate of asymptomatic carriage followed the same pattern than the attacks incidence, with an approximately three month shift though.

Interestingly this shift disappeared in 2005, when chloroquine was replaced by amodiaquine and when asymptomatic malaria cases where systematically treated, giving rise to the complete overlapping of asymptomatic carriage rate and attacks incidence (Figure 4).

**Figure 5:**
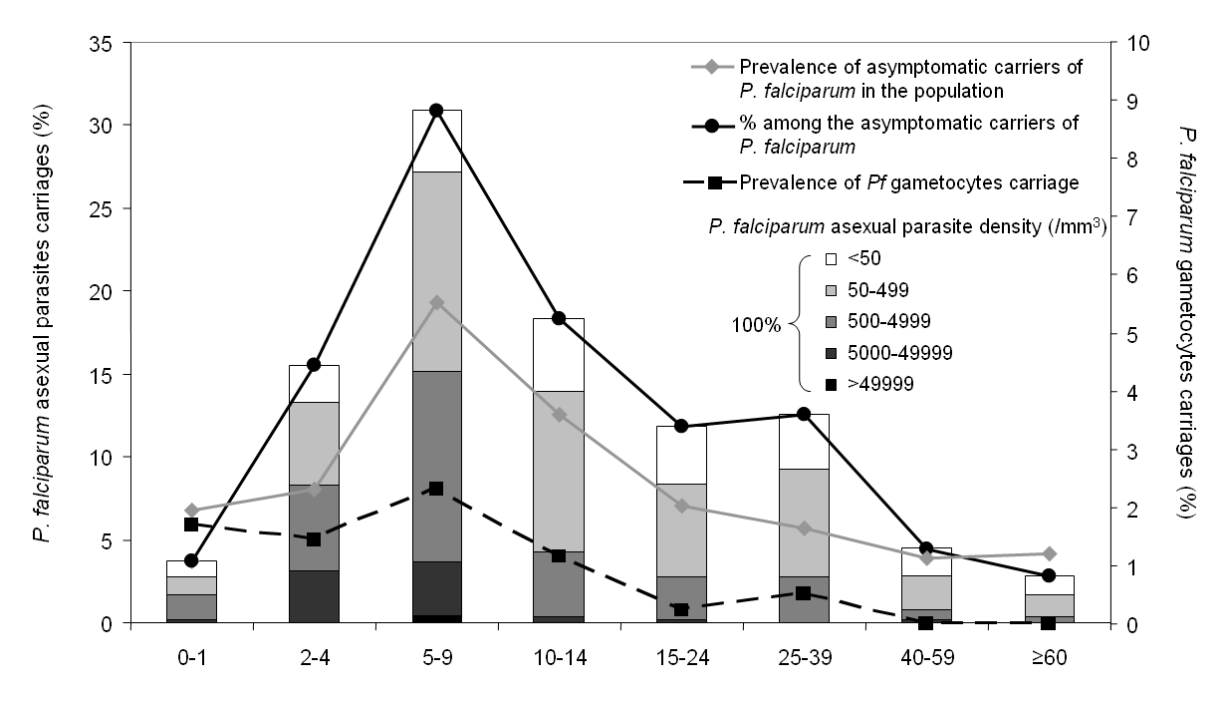
**Asymptomatic carriage of *P. falciparum *parasites according to age**. Data come from the monthly malaria active detection. Age distribution of symptomatic carriage among positive cases during systematic sampling (black circle) and parasite densities (histograms) in the 541 cases of asymptomatic carriage of *P. falciparum *observed from February 2003 to June 2005. Prevalence of asymptomatic carriages (grey rhombus) is calculated by the ratio between positive slides for *P. falciparum *and total number of slides (from active detection cases in the absence of fever). Among the 541 positive slides, 57 (10.53%) had *P. falciparum *gametocytes (black square). For each sample, the age of the subject used was the age at the time of the blood intake.

Prevalence of asymptomatic *P. falciparum *malaria was at highest level (12.3%) in children from five to nine years old (Figure 5). Fourteen percent of asymptomatic children under 10 years displayed high parasite loads (density over 5,000 parasites/μl) versus only 1.5% of the older asymptomatic parasite carriers (Figure 5). In the same time, levels of *P. falciparum *gametocytes were irregular and did not follow a seasonal variation at all. Only 10.5% (n = 57) of smears were positive for gametocytes and remarkably this essentially concerned children under 15 years old (86%; χ^2 ^test; p < 0.001). This highlights that children and teenagers constituted the principal reservoir of infective parasites. Altogether those data indicated that profiles of asymptomatic malaria cases seemed to follow profiles of malaria attack cases, in term of seasonality, prevalence of *P. falciparum *infection and children sensibility.

## Discussion

The tight relationships existing between malaria epidemiology and land altitude have been extensively studied both in mountain and costal regions [12,13]. In this Saharevo project, the longitudinal study performed among villagers described with accuracy the seasonality and the intensity on *P. falciparum *infection there. This survey, based on the analysis of data collected over 10 years, enables in-depth determination of malaria epidemiology in areas of intermediate altitude in order to set up adapted strategy in accordance with current malaria elimination policies in Madagascar.

### Introducing a vector control plan in the eastern foothills of Madagascar

Saharevo is located in an intermediate zone in between coastal areas with high-transmission equatorial malaria and highland areas with unstable malaria transmission but malaria epidemic risks [1]. In term of malaria epidemiological patterns, this village displays mesoendemic malaria. Strong seasonal variations of malaria incidence indicate that malaria transmission increases from January – when rainfalls are maximal, and peaks in April – coincident with rainy season completion, before getting back close to zero level. Coincidence between transmission peak and rainy season is related to vectors density and behavior. This is supported by the fact that April is also the season of paddies water-filling, multiplying surfaces for hosting *An. funestus *breeding sites [14], the major malaria vector in Saharevo. Thus, altogether, rice field proximity, altitude, and economic status influence malaria prevalence in surrounding communities [15-17].

Before and during the time of this project, no interventions to control vectors have been conducted in the region, since this was not part of the national the national policy for foothills areas at that time. The annual EIR in Saharevo was estimated at 2.78 in 2004 and this indicates a low malaria transmission in the foothills areas of Madagascar, at least when compared to EIR in coastal areas in Madagascar or in other areas in mainland Africa [18,19]. By contrast, despite the weak transmission, parasitological data presented herein are consistent with a relatively high level of malaria endemicity in Saharevo (Figure 2). As in many rural areas in Madagascar, the poor quality of traditional houses in Saharevo promotes the in and out journey of mosquitoes at ease, especially since no vector control measures have ever been implemented in this zone for more than a decade including the project period. On top of that, the villagers currently display a low immunity against malaria; hence most of mosquito bites are likely to lead to an infection (malaria with or without clinical signs). From our previous study carried out in Mahasolo, located in the western foothills area of Madagascar, a similar high malaria endemicity was also described despite a low malaria transmission level [20]. This particular malaria epidemiological profile in the foothills of Madagascar could be controlled by use of indoor spraying of insecticide and insecticide treated bed nets. Moreover, the success of the vector control in the foothills areas in Madagascar has been proven and admitted since the 1950s [7,21]. Thus, pursuing efforts in vector control activities will significantly help reducing malaria transmission intensity, malaria incidence and malaria prevalence among population in Saharevo in particular, and in extended foothill areas in general. Therefore, it is clearly stated in the national strategy for malaria control revised in April 2009 that all activities will be strengthened from 2009 to 2012 to achieve malaria elimination in Madagascar. Scaling-up the vector control based on the use of insecticide-treated bed nets and the indoor spraying of insecticides is on top priority.

### Introducing new malaria case management in the eastern foothills of Madagascar

Until the final year of the project, *P. falciparum *was predominant in Saharevo (98% of malaria cases). Chloroquine has been used as the first line treatment and the failure rate to this treatment was more than 15% as reported herein or elsewhere [7,22,23]. Even though there was no early treatment failure and mainly late parasitological failures that occurred after day 7, this indicates a limited therapeutic efficacy of this drug. It is only in 2005 that amodiaquine was used. Amodiaquine has revealed its high efficacy in this study which has been used as a reference to lead changing in treatment policy in Madagascar [22].

The incidence of malaria attacks was the highest in two to four years old children although the prevalence of asymptomatic carriers preferentially affected the five to nine years old group. Before amodiaquine introduction, prevalence of asymptomatic carriers tent to peak three months later than incidence of malaria attacks. This supports that a former malaria attack offers a better tolerance to face further infections. When amodiaquine was used to treat malaria infection in Saharevo, this shift immediately disappeared simultaneously with a minor proportion of asymptomatic carriers (Figure 4), although no significant reduction of malaria incidence could be really observed. This highlights the rapid effect on malariometric parameters following a changing in drug policy. The importance of adopting an efficient treatment was also illustrated. The benefits of amodiaquine use will be definitely implemented with the deployment of the artemisinin-based combination therapy artesunate + amodiaquine. It is worth mentioning that recent articles report how efficacy this combination is, especially in Madagascar [24,25].

From these observations, drugs clearly play a pivotal role to control malaria and especially to turn towards malaria elimination in Madagascar. Interest of introducing the artesunate + amodiaquine combination relies also on its gametocytocide activity. While 86% of gametocyte carriers were < 15 year old children, forming the main parasite reservoir in Saharevo, such a policy would have an impact on malaria transmission especially in children. At the community level – especially in remote villages in foothill areas, replacing pre-packaged chloroquine by the artesunate + amodiaquine formulation for in-home treatment during rainy season should be a successful approach to ensure an efficient reduction of malaria transmission.

### Improving malaria diagnosis in the eastern foothills of Madagascar

In Saharevo, almost 10% of malaria attacks occur with very low parasite load less than 200 parasites/μl. Nevertheless, co-infections with viruses or other pathogens might simultaneously occur. The parasite density required to distinguish malaria attacks from other fever causes is unclear in the context of low transmission intensity [26]. Moreover, diagnosing co-infections and establishing pyrogenic thresholds would be strongly compromised by both practical and public health issues anyway. Most of Malagasy primary health centers can not run diagnosis for bacterial or viral infections due to a lack of equipments and resources. Also, practically, from a public health angle, there is a limited interest to determine malaria pyrogenic threshold especially in regions with seasonal transmission as it is in Saharevo and in Madagascar generally. This point is particularly relevant in the context of malaria elimination policy. Since the transition period preceding proper malaria elimination is expected to be marked by a drop of malaria prevalence and incidence, an increased proportion of malaria attacks with low parasitaemia are expected among the remaining cases both in children and adults, due to the loss and the absence of acquired immunity in the population. Then, *Plasmodium *sp tracking and screening have to become fundamental aspects for achieving malaria elimination, whilst any malaria case detected has to be treated irrespective of the parasitaemia. This is expected to contribute to the reduction of malaria transmission.

During the malaria transmission season (January – July), almost half of the malaria-presumed fevers were confirmed cases of malaria attacks by microscopy in Saharevo. This even dropped to less than a quarter of them during the low malaria transmission season (August – December). On that basis, in the integrated management of childhood illnesses at the community level or at a heath centres that do not use malaria biological diagnosis, more than half of the cases of fevers may be treated abusively with antimalarial. Providing tools for biological diagnosis of malaria to discriminate non-malaria cases will be crucial to develop case management programs in areas with a malaria epidemiologic context similar to that of Moramanga where Saharevo is. Microscopy is a better choice for health facilities, but rapid diagnostic test can be routinely used at the health facilities and at the community level. With regards to rapid diagnostic test, from recent experiences in Madagascar, detecting pLDH will be helpful for this purpose [27] since the remanence of pfHRP will increase the proportion of the false positive results [28].

### Towards the application of malaria elimination and the strengthening of malaria control strategies in Madagascar

In Madagascar, importance of studying malaria epidemiology in foothills located at 800 to 1,000 m above the sea relies on that a third of the Malagasy population (approximately 6,623,800 inhabitants) live nowadays in such area. In accordance with the major results of our studies, the MoH plans to extend the vector control to the foothills. Malaria endemicity in intermediate areas is then expected to drop and foothills will progressively become regions prone to malaria epidemics, closer to highlands. This may imply changing in malaria trends while children under five and pregnant women will not be the only vulnerable groups. In this context, lessons from the past would help to adjust strategies in response to the malaria epidemiology evolution. As mentioned above, former success of malaria prevention through DDT indoor spraying occurred between 1940s and 1960s, especially in the highlands and the foothill areas in Madagascar [7,21]. The sudden interruption of the DDT indoor spraying was a lot responsible of the fatal malaria epidemic in the 1980s that triggered deaths mainly in these regions [7,29]. From this gloomy experience is highlighted how crucial it is to keep sustaining both cure and prevention to combat malaria. Madagascar can also learn from the experiences in other countries that reached a significant malaria reduction for making malaria policy change successful [30-33].

## Concluding remarks

In Saharevo, malaria transmission is seasonal. The incidence of malaria is important among children but slightly decreases with age. These results support that a premunition against malaria is slowly acquired until adolescence although this premunition remains weak premunition in villagers and by extension in the whole eastern foothill area of Madagascar.

From a public health angle, the Saharevo experience nicely illustrates once more that an adequate malaria care, with a confirmed diagnosis and early effective treatment prevent deaths. This must be routinely extended to the whole foothills and to the entire country in Madagascar. These foothill areas are potentially considered as "the entrance gate" of malaria parasites from low land (with high transmission) to highlands (with low transmission). For instance, the use of fixed-dose combination of artesunate + amodiaquine to treat malaria in the primary health centres, improved community case management of malaria in remote areas – which has been already part of the Malagasy malaria control policy [34], combined with an active vector control plan, should be the efficient partners to tackle directly malaria endemicity in the foothills and indirectly epidemics in the highlands. As soon as vector control will be initiated, regular surveys will be fundamental (i) to up-date malariometric indexes including parasite rate among villagers, uncomplicated malaria cases at the primary health centres, and severe malaria trends at the hospitals; and (ii) to adjust the local strategies for an optimal application of the national policy.

## Competing interests

The authors declare that they have no competing interests.

## Authors' contributions

FA, OD, VR, LAR, JBD, RJ, and MR prepared the study design. OD, LPR, and FA contributed to data analysis. LAR, AR, LR, LAR, FR, RR, JR, MAR, and JR performed field and laboratory work. OD, VR, FA, and MR drafted and critically revised the manuscript. All authors read and approved manuscript. MR, OD, VR, and FA are guarantors of this paper.
